# Supplementary material for: Optimizing the Construction of Outcome Measures for Impact Evaluations of Intimate Partner Violence Prevention Interventions
Source: J Interpers Violence. 2023 Apr 9;38(15-16):9105–31. doi: 10.1177/08862605231162887 (PMC10668532; doi:10.1177/08862605231162887)
Supplement: sj-docx-1-jiv-10.1177_08862605231162887 – Supplemental material for Optimizing the Construction of Outcome Measures for Impact Evaluations of Intimate Partner Violence Prevention Interventions [file sj-docx-1-jiv-10.1177_08862605231162887.docx]

Appendix Table 3: Description of Studies

|  |  |  |  |  |  |  | **IPV Prevalence in Endline Control** | | **Baseline** | | |  |
| --- | --- | --- | --- | --- | --- | --- | --- | --- | --- | --- | --- | --- |
| Name | Study design | Sample Size | Setting | Population enrolled | Programme description | Group size for sessions | 12-month physical IPV, women's reports | 12-month sexual IPV, women's reports | Mean Age | Percent who did not complete primary education | Household SES | Percent married or partnered/cohabiting |
|  |  |  |  |  |  |  | % | % | % | % |  |  |
| **Bandebereho** Doyle et al, 2018 | RCT, with individual randomization | 1195 men | Rural Rwanda | Married or cohabitating men, 21 to 35, expecting or with a child under 5, and their female partners | 15 session participatory curriculum for new fathers; female partners were invited to 8 of the sessions | 12 couples | 56.5 | 60.2 | 28.7 men 26.6 women | 62.8 men | 38.7 % of households report being able to afford basic household goods never or only sometimes | 100 |
| **Becoming One** Boyer et al, forthcoming | RCT, with individual randomization | 1680 couples | Rural Uganda | Married couples 18 to 65 who had been together at least one year | 12 session, participatory curriculum for couples, facilitated by faith leaders | maximum 7 couples (14 people) | 23.8 | 32 | 36.5 men 31.6 women | 58.5 men 76.8 women | 22.4% in lowest wealth quintile, 40.9% below $1.90/day PPP, 47.3% report concerns about having enough food in past month | 100 |
| **Indashyikirwa.** Dunkle et al 2020 | Cluster RCT; couples followed longitudinally | 1660 couples | Rural Rwanda | Married or cohabitating partners, 15 to 49, where at least one member was an active VSLA member | 21 session couples’ curriculum; 16 additional sessions on activism skills with subset of couples; Creation and staffing of women's safe spaces; Opinion Leader Training | maximum 15 couples (30 people) | 32.3 | 38.1 | 35.4 men 32.5 women | 14.8 men 18.4 women | Household weighted asset score: 7.0 (0-15.2) | 100 |
| **Maisha CRT01** Kapiga et al 2018 | Cluster RCT - randomized by loan group | 919 women | Secondary city, Tanzania | Low income women enrolled in existing BRAC microfinance loan groups | 10 session Maisha partipatory curriculum to empower women and reduce IPV | 20 (16-24) | 19 | 17 | 39.5 women | 14.3 women | 47 % report economic hardship in last year; Median monthly income 220,000 TS (US$ 84) | 88 |
| **Maisha CRT02** Harvey et al, 2021 | Cluster RCT - randomized by newly formed neighborhood groups | 1126 women | Secondary city, Tanzania | Women, aged 20 to 50, who were not a member of a loan group and not formally employed | 10 session Maisha participatory curriculum to empower women and reduce IPV | unclear | 20 | 21 | 33 women | 19 women | 64.7 report economic hardship in last year; Median monthly income 110,000 TS (US$47) | 91 |
| **Stepping Stones/Creating Futures.** Gibbs et al, 2020 | Cluster RCT | 13 | Urban informal community, Johannesburg | Young people, ages 18 to 30, not in education or employment | 10 sessions (Stepping Stones) plus 11 session (Creating Futures) each ~3 hours long, delivered twice a week to single sex groups | ~20 | 44 | 27.2 | 23.8 | ~11 men and ~8% completed primary school only | 37% of young men and 25% of young women stole in the last week because of hunger | 64 |
| **Unite for a Better Life.** Sharma et al, 2020 | Cluster RCT -64 villages randomly selected and then randomly allocated to 4 arms: men only; women only; couple's group; and comparison. Within each village, 106 eligible households were randomly selected for inclusion in the trial. In the 3 intervention arms, 80% of enrolled households were individually randomized to receive the intervention, the other 20% were included in baseline and endline data collection to assess spillovers. | 1691 men enrolled in men's UBL group and their wives 1692 in the control arm | Rural Ethiopia | Households with married or cohabitating couples where the woman was 18 to 49 were eligible to participate. | 14 participatory gender and HIV sessions delivered twice weekly during Ethiopian coffee ceremony | 20 | 20.1% of wives among Men's UBL group | 37.4% of wives among Men's UBL group | 17.1% of men and 37.1% of wives in Men's UBL group were less than 30 years old | 42.8% of men had no schooling; 52.4% completed primary in the Men's UBL group  76.9% of wives had no schooling; 21.6% completed primary in the Men's UBL group | 63.8% of HH are in poorest wealth quintile | 100 |

Appendix table 4: Items used to measure IPV across studies

| ***Bandebereho- women*** |  |
| --- | --- |
| *Physical IPV* |  |
| In the past 12 months, how many times has your current husband (1) slapped you or thrown something at you which could hurt you; (2) pushed or shoved you; (3) hit you with a fist or with something else which could hurt you; (4) kicked, dragged, beaten, choked or burnt you; (5) threatened to use or actually used a knife or stick against you? | Never, once, a few times, frequently |
| *Sexual IPV* |  |
| In the past 12 months, how many times has your husband (1) physically forced you to have sex with him when you didn’t want to; (2) consented to sex out of fear of what your partner might do if you refused? | Never, once, a few times, frequently |
| *Emotional IPV* |  |
| In the past 12 months, how many times has your current husband (1) insulted you or made you feel bad about yourself (2) belittled or humiliated you in front of other people (3) verbally threatened to hurt you or someone you care about (4) done things to scare or intimidate you on purpose (e.g. by the way he looked at you, by yelling and smashing things)? | Never, once, a few times, frequently |
| ***Becoming One – women*** |  |
| *Emotional IPV* | *Response Categories* |
| In the past 12 months, how many times has your partner (1) said or done something to humiliate you in front of others? (2) threatened to hurt or harm you or someone you care about?; (3) insulted you or made you feel bad about yourself | Never, once, a few times, many times |
| *Physical IPV* |  |
| In the past 12 months, how many times has your partner (1) slapped you; (2) pushed you, shook you, or thrown something at you which could hurt you; (3) punched you with a fist or with something else which could hurt you; (4) kicked, dragged, or beat you up; (5) twist your arm or pull your hair? (6) tried to choke or burn you on purpose? (7) threatened to use or actually used a gun, knife or other weapon against you? | Never, once, a few times, many times |
| *Sexual IPV* |  |
| In the past 12 months, how many times has your partner (1) physically forced you to have sex with him when you didn’t want to?; (2) threaten or attempt to coerce you in any other way to perform sexual acts you did not want to?; (3) used physical force or threats to make you do something else sexual that you did not want to do? | Never, once, a few times, many times |
| ***Indashyikirwa- women*** |  |
| *Emotional IPV* | *Response Categories* |
| In the past 12 months, how many times has your current husband (1) insulted you or made you feel bad about yourself (2) belittled or humiliated you in front of other people (3) verbally threatened to hurt you or someone you care about (4) done things to scare or intimidate you on purpose (e.g. by the way he looked at you, by yelling and smashing things)? | Never, once, a few times, many times |
| *Physical IPV* |  |
| In the past 12 months, how many times has your current husband (1) slapped you or thrown something at you which could hurt you; (2) pushed or shoved you; (3) hit you with a fist or with something else which could hurt you; (4) kicked, dragged, beaten, choked or burnt you; (5) threatened to use or actually used a gun, knife or other weapon against you? | Never, once, a few times, many times |
| *Sexual IPV* |  |
| In the past 12 months, how many times has your husband (1) physically forced you to have sex with him when you didn’t want to; (2) used threats or intimidation to make you have sex when you did not want to; (3) used physical force or threats to make you do something else sexual that you did not want to do? | Never, once, a few times, many times |
| ***Indashyikirwa- men*** |  |
| *Physical IPV* |  |
| In the past 12 months, how many times have you (1) slapped your wife or thrown something at her that could hurt her; (2) pushed or shoved your wife; (3) hit your wife with a fist or with something else which could hurt her; (4) kicked, dragged, beaten, choked or burnt your wife on purpose; (5) threatened to use or actually used a gun, knife or other weapon against your wife? | Never, once, a few times, many times |
| *Sexual IPV* |  |
| In the past 12 months, how many times have you (1) physically forced your wife to have sex with you when she didn’t want to; (2) used threats or intimidation to get your wife to have sex when she did not want to; (3) used physical force or threats to make your wife do something else sexual that you she not want to do? | Never, once, a few times, many times |
| ***MAISHA women*** |  |
| *Physical IPV* |  |
| In the past 12 months, has your current partner: 1) slapped you or threw something at you that could hurt you; 2) pushed or shoved you or pulled your hair; 3) hit you with a fist or with something that could hurt you; 4) kicked you, dragged you, or beat you up; 5) choked or burned you on purpose; 6) threatened to use or actually used a gun, knife, or other weapon against you. | No, yes |
| *Sexual IPV* |  |
| In the past 12 months, has your current or other partner:1) physically force you to have sexual intercourse by threatening you, holding you down or hurting you in some way; 2) had sexual intercourse because you were afraid that your partner would hurt you or someone you cared about if you refused; 3) did you have sexual intercourse you did not want to because you were afraid that your partner would leave or take another girlfriend if you refused? | No, yes |
| *Emotional IPV* |  |
| In the past 12 months, has your current or other partner: 1) insulted you or made you feel bad about yourself: 2) belittled or humiliated you in front of other people; 3) done things to scare or intimidate you on purpose (for example, by the way he looked at you, by yelling, by smashing things); 4) verbally threatened to hurt you or someone you care about. | No, yes |
| ***Stepping Stones Creating Futures- men*** |  |
| *Emotional IPV* | *Response Categories* |
| In the past 12 months, how many times have you (1) insulted a partner or deliberately made her feel bad about herself (2) belittled or humiliated a partner in front of other people (3) done things to scare or intimidate a partner on purpose for example by the way you looked at her, by yelling and smashing things (4) threatened to hurt a partner | Never, once, a few times, many times |
| *Physical IPV* |  |
| In the past 12 months, how many times have you (1) slapped your current or previous girlfriend or wife or thrown something at her that could hurt her; (2) pushed or shoved your current or previous girlfriend or wife; (3) hit your current or previous girlfriend or wife with a fist or with something else which could hurt her; (4) kicked, dragged, beaten, choked or burnt your current or previous girlfriend or wife on purpose; (5) threatened to use or actually used a gun, knife or other weapon against your current or previous girlfriend or wife? | Never, once, a few times, many times |
| *Sexual IPV* |  |
| In the past 12 months, how many times have you (1) physically forced your current or previous girlfriend or wife to have sex with you when she didn’t want to; (2) used threats or intimidation to get your current or previous girlfriend or wife to have sex when she did not want to; (3) used physical force or threats to make your current or previous girlfriend or wife do something else sexual that you she not want to do? | Never, once, a few times, many times |
| ***United for a Better Life- women*** |  |
| *Physical IPV* |  |
| In the past 12 months, has your husband ever: 1) slapped you or threw something at you that could hurt you; 2) pushed or shoved you; 3) hit you with a fist or with something that could hurt you; 4) kicked you, dragged you, or beat you up; 5) choked or burned you on purpose; 6) threatened to use or actually used a gun, knife, or other weapon against you. | No, yes |
| *Sexual IPV* |  |
| In the past 12 months, has your husband ever:1) physically force you to have sexual intercourse with him even when you did not want to; 2) force you to perform sexual acts that you did not want to; 3) did you ever have sexual intercourse because you were intimidated by him or afraid he would hurt you? | No, yes |
| *Emotional IPV* |  |
| In the past 12 months, has your husband ever: 1) insulted you or made you feel bad about yourself: 2) belittled or humiliated you in front of other people; 3) done things to scare or intimidate you on purpose (for example, by the way he looked at you, by yelling, by smashing things); 4) threatened to hurt you or someone you care about. | No, yes |
| ***United for a Better Life- men*** |  |
| *Physical IPV* |  |
| In the past 12 months have you ever: 1) slapped your wife or thrown something at her that could hurt her; 2) pushed or shoved her; 3) hit her with a fist or with something that could hurt her; 4) kicked her, dragged her, or beat her up; 5) choked or burned her on purpose; 6) threatened to use or actually used a gun, knife, or other weapon against her. | No, yes |
| *Sexual IPV* |  |
| In the past 12 months have you ever: 1) physically forced your wife to have sexual intercourse with you even when she did not want to; 2) forced her to perform sexual acts that she did not want to; 3) did your wife ever have sexual intercourse because she was intimidated by you or afraid you would hurt her? | No, yes |
| *Emotional IPV* |  |
| In the past 12 months have you ever: 1) insulted your wife or made her feel bad about herself; 2) belittled or humiliated her in front of other people; 3) done things to scare or intimidate her on purpose (for example, by the way you looked at her, by yelling, by smashing things); 4) threatened to hurt her or someone she cares about. | No, yes |
